# Tunable Electromagnetically and Optomechanically Induced Transparency in a Spinning Optomechanical System

**DOI:** 10.3390/e28030324

**Published:** 2026-03-13

**Authors:** Haoliang Hu, Jinting Li, Xiaofei Li, Han Wang, Haoan Zhang, Yue Yang, Shanshan Chen, Shuhang You

**Affiliations:** 1 China Electric Power Research Institute Wuhan Branch, Wuhan 430074, China; 2 Innovation Academy for Precision Measurement Science and Technology, Chinese Academy of Sciences, Wuhan 430071, China

**Keywords:** optomechanics, electromagnetically induced transparency, optomechanically induced transparency, sagnac effect, atomic ensemble

## Abstract

We investigate the optical response properties of an atom-assisted spinning optomechanical system, in which a spinning optical resonator is coupled simultaneously to a two-level atomic ensemble and a mechanical resonator driven by a weak pump field. Remarkably, we demonstrate that by simply reversing the rotation direction, the system can be switched between a low-absorption electromagnetic and optomechanically induced transparency state and a high-absorption state, constituting a form of non-reciprocal optical control at the quantum level. Furthermore, by tuning the phase difference between the mechanical pump and the probe field, direction-dependent switching between absorption and gain is achieved. These non-reciprocal effects originate from the Sagnac-induced frequency shift in the optical mode, which leads to distinct optomechanical and atom–cavity couplings for opposite spinning directions. We also show that the absorption spectrum can be modulated by the angular velocity and the atomic number. Our results indicate that the optical properties of the hybrid system can be manipulated via the angular velocity, phase difference, and atom number, with potential applications in chiral photonic communications.

## 1. Introduction

Electromagnetically induced transparency (EIT) is an effect based on quantum interference in a three-level atomic system. Through the coherent interaction between coupling light and probe light, a controllable transparency window is created in the medium, accompanied by strong dispersion [1,2]. After the EIT effect based on the atomic system was observed [3], its distinctive applications in both nonlinear optics and optical (quantum) information processing have drawn substantial attention [4,5,6,7,8]. Inspired by the theoretical framework of EIT, researchers have proposed a quantum interference effect named optomechanically induced transparency (OMIT), which arises from the interaction between light and a mechanical resonator in an optomechanical system (OMS) [9]. This phenomenon has shown great potential in optical storage [10,11], optical switching [12,13] and precision measurement [14,15,16], driving interdisciplinary research at the frontier of quantum sensing and micro/nanophotonics.

An OMS is a platform where cavity fields and mechanical resonators are coupled via radiation-pressure forces, serving as a crucial bridge between the classical and quantum worlds [17]. Significant progress has been made in the study of OMIT within OMS [18,19,20,21,22]. For example, an OMS consisting of a Λ-type three-level atom, a mechanical resonator, and a sideband-driven cavity is proposed, and electromagnetic and optomechanically induced transparency (EOMIT) and amplification (EOMIA) phenomena are demonstrated [18]. The effect of the control field phase on the output field propagation and Stokes field generation in a ring cavity optomechanical system is studied, and a tunable group delay is achieved [19]. The optical response of a hybrid optodynamic system with secondary coupling is theoretically analyzed, and adjustable optical transparency and amplification effects are achieved by adjusting the gain rate of the auxiliary cavity and other parameters [20].

In hybrid OMS, various novel quantum phenomena can emerge, such as quantum state transfer [23,24], tunable slow and fast light [25,26,27,28], and optical bistability [29,30]. Therefore, investigating the quantum properties of hybrid OMS is an inevitable trend for advancing research in quantum optics. Recently, significant progress has been achieved in atom-assisted OMS [11,31,32,33,34,35]. For example, vibration-induced transparency and its application in micro-mass sensing are investigated by simulating photomechanical systems with movable atoms [31]. Other advancements include enhanced OMIT realized via atomic ensembles in OMS [11], as well as atom-assisted coherent control of multiple-color mechanically induced switching [32]. Thus, the study of the optical response characteristics of atom-assisted cavity OMS is crucial for providing important theoretical guidance in controlling optical transmission characteristics.

Furthermore, a rotating optomechanical resonator can introduce direction-dependent frequency shifts in the optical mode due to the Fizeau shift [36,37,38]. This, in turn, modifies the coupling strength between optical and mechanical modes, thereby altering the system’s quantum properties [39,40,41,42]. These changes may further enable novel control over quantum state transfer, entanglement generation, and noise suppression in rotating OMS.

In this paper, we present a theoretical model for an atom-assisted rotating OMS to study the optical response characteristics of the hybrid system. Specifically, a rotating optical resonator is simultaneously coupled to a two-level atomic ensemble and a weakly pumped mechanical resonator. The rotational motion induces a frequency shift in the optical mode, which leads to different optomechanical coupling strengths and atom–cavity interaction intensities for different spinning directions. This work touches upon several themes central to the study of quantum dynamics in hybrid systems, including light–matter interactions arising from the coupling between the cavity field and both the atomic ensemble and the mechanical resonator, hybrid systems bridging distinct physical domains by combining photonics, atomic physics, and nanomechanics, and quantum dynamics manifested in the direction-dependent optical response and quantum interference effects.

## 2. Theoretical Model

**Figure 1:**
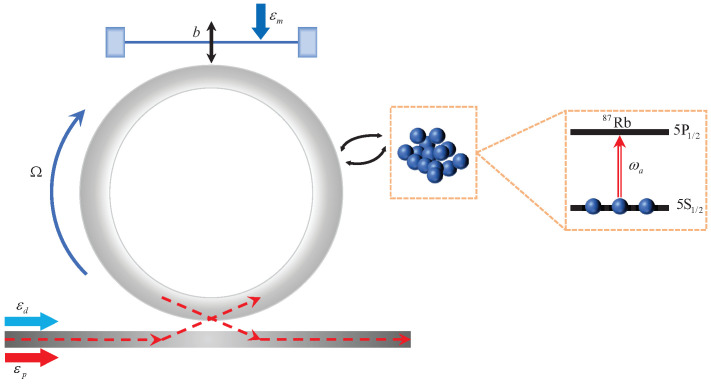
The schematic of the atom-assisted optomechanical system based on a spinning whispering-gallery resonator.

In this scheme, as shown in Figure 1, we consider an atom-assisted optomechanical system consisting of a spinning whispering-gallery-mode (WGM) resonator, a mechanical oscillator and a cold Rb87 atom of a two-level atom. The two-level quantum states |0〉 and |1〉 of the atom correspond to the quantum states $|5S_{1/2}\rangle$ and $|5P_{1/2}\rangle$ of the $D_{1}$ line of Rb87, respectively. The spinning WGM resonator is driven by the pump field at frequency $\omega_{d}$ and the probe field at frequency $\omega_{p}$, with the mechanical resonator excited by a weak mechanical pump field of frequency $\omega_{m}$ in this atom-assisted hybrid system. The amplitudes of the pump and probe fields with powers $P_{d}$ and $P_{p}$, respectively, are $\varepsilon_{d}=\sqrt{2\kappa P_{d}/\left(\hbar \omega_{d}\right)}$ and $|\varepsilon_{p}|=\sqrt{2\kappa P_{p}/\left(\hbar \omega_{p}\right)}$, respectively. κ is the optical loss rate. Owing to the Sagnac effect, the optical mode undergoes a Sagnac–Fizeau shift [43], namely, $\omega_{c}\to \omega_{c}+\Delta_{F}$ where $\Delta_{F}$ is given by

(1) $$\begin{array}{c} \Delta_{F}=\frac{nr\Omega \omega_{c}}{c}\left(1-\frac{1}{n^{2}}-\frac{\lambda }{n}\frac{dn}{d\lambda }\right), \end{array}$$

Here, $\omega_{c}$ represents the optical resonance frequency of the stationary WGM resonator; *n* and *r* denote the refractive index and radius of the WGM resonator, respectively. In a vacuum, *c* stands for the speed of light, and λ denotes its wavelength. Ω is the angular rotation speed (positive for clockwise rotation). The dispersion term $\frac{dn}{d\lambda }$ describes the relativistic origin of the Sagnac effect and is relatively small in typical materials (∼1%). $\Delta_{F}>0$ ($\Delta_{F}<0$) corresponds to Ω>0 (Ω<0), i.e., the light propagates against (along) the rotation of the spinning WGM resonator.

The Hamiltonian of the system can be written as

(2) $$\begin{array}{c} H=H_{0}+H_{I}+H_{D}, \end{array}$$

with

(3) $$\begin{array}{ccc} H_{0} & = & \hbar \left(\omega_{c}+\Delta_{F}\right)c^{†}c+\hbar \omega_{a}a^{†}a+\hbar \omega_{b}b^{†}b,\\ H_{I} & = & -\hbar g_{1}\sqrt{N}\left(ca^{†}+c^{†}a\right)-g_{2}c^{†}c\left(b+b^{†}\right),\\ H_{D} & = & i\hbar \varepsilon_{d}\left(c^{†}e^{-i\omega_{d}t}-ce^{i\omega_{d}t}\right)+i\hbar \left(c^{†}\varepsilon_{p}e^{-i\omega_{p}t}-c\varepsilon_{p}^{*}e^{i\omega_{p}t}\right)\\ & + & i\hbar (b^{†}\varepsilon_{m}e^{-i\omega_{m}t}-c\varepsilon_{m}^{*}e^{i\omega_{m}t}). \end{array}$$

Here *c* ($c^{†}$) and *b* ($b^{†}$) are the annihilation (creation) operators for the optical mode and the mechanical mode, respectively. $\omega_{a}$ is the energy-level spacing between the two atomic states |0〉 and |1〉. $\omega_{b}$ is the resonance frequency of the mechanical resonator. $H_{0}$ is the free Hamiltonian of the system. $H_{I}$ describes the interaction energy between the atomic ensemble and the optical mode, as well as that between the optical mode and the mechanical mode. *N* is the total number of atoms in the atomic ensemble. For weak excitation and N≫1, we can define the collective operator of the atomic ensemble $a=\left(1/\sqrt{N}\right)\sum_{i=1}^{N}{|0\rangle }_{ii}\langle 1|$ [44,45,46], which satisfies the bosonic commutation relation $[a,a^{†}]=1$. The factor $\sqrt{N}$ in the interaction Hamiltonian accounts for the collective enhancement due to the atomic ensemble. $g_{1}=\mu \sqrt{\omega_{c}/\left(2\hbar V\epsilon_{0}\right)}$ is the coupling coefficient between the cavity field and a single atom, where μ is the electric dipole moment associated with the atomic transition, *V* is the volume of the WGM resonator, and $\epsilon_{0}$ is the permittivity of vacuum. The optical mode *c* is coupled to the mechanical mode via the radiation-pressure interaction, with a single-photon coupling strength $g_{2}=\left(\omega_{c}/r\right)\sqrt{\hbar /(2m\omega_{b})}$, where *m* denotes the mass of the mechanical resonator. $H_{D}$ represents the external laser driving the optical mode and mechanical mode. The driving field amplitudes are defined as $\epsilon_{p}=\left|\epsilon_{p}\right|e^{i\phi_{p}}$ and $\epsilon_{m}=\left|\epsilon_{m}\right|e^{i\phi_{m}}$, where $\phi_{p}$ and $\phi_{m}$ are the initial phases of the probe field and the mechanical pump field, respectively. The phase difference between these two fields is defined as $\Phi =\phi_{m}-\phi_{p}$, which appears in Equation (10) and plays a crucial role in controlling the quantum interference as discussed in Figure 4.

In a rotating frame with the rotation operator $U=\exp[-i\omega_{d}\left(a^{†}a+c^{†}c\right)]$, the Heisenberg-Langevin equations of motion of the system can be calculated as

(4) $$\begin{array}{ccc} \dot{a} & = & -\left[\gamma_{a}+i\left(\omega_{a}-\omega_{d}\right)\right]a+ig_{1}\sqrt{N}c+\sqrt{\gamma_{a}}a_{in},\\ \dot{b} & = & -\left(\gamma_{m}+i\omega_{b}\right)b+ig_{2}c^{†}c+\varepsilon_{m}e^{-i\omega_{m}t}+\sqrt{2\gamma_{m}}b_{in},\\ \dot{c} & = & -\left[2\kappa +i\left(\omega_{c}+\Delta_{F}-\omega_{d}-g_{2}\left(b+b^{†}\right)\right)\right]c+\varepsilon_{d}\\ & + & \varepsilon_{p}e^{-i\delta t}+ig_{1}\sqrt{N}a+\sqrt{2\kappa }c_{in}, \end{array}$$

where $\delta =\omega_{p}-\omega_{d}$ is the frequency difference between the probe field and the driving field, $\gamma_{a}$ ($\gamma_{m}$) is the decay rate of the atomic ensemble (mechanical mode). $a_{in}$, $b_{in}$ and $c_{in}$ are the noise operators for the two-level atoms, the mechanical mode and the optical mode, respectively, which have zero mean values $\langle a_{in}\rangle =\langle b_{in}\rangle =\langle c_{in}\rangle =0$ and satisfy the standard correlation functions in the Langevin formalism.

We denote 〈a〉, 〈b〉 and 〈c〉 as the expectation values of the operators *a*, *b* and *c*, respectively. The time evolution of these expectation values is given by

(5) $$\begin{array}{ccc} \langle \dot{a}\rangle & = & -\left(\gamma_{a}+i\Delta_{a}\right)\langle a\rangle +ig_{1}\sqrt{N}\langle c\rangle ,\\ \langle \dot{b}\rangle & = & -\left(\gamma_{m}+i\omega_{b}\right)\langle b\rangle +ig_{2}\langle c^{†}\rangle \langle c\rangle +\varepsilon_{m}e^{-i\delta t},\\ \langle \dot{c}\rangle & = & -\left[2\kappa +i\left(\Delta_{0}+\Delta_{F}-g_{2}\left(\langle b\rangle +\langle b^{†}\rangle \right)\right)\right]\langle c\rangle \\ & + & \varepsilon_{d}+\varepsilon_{p}e^{-i\delta t}+ig_{1}\sqrt{N}\langle a\rangle , \end{array}$$

where $\Delta_{a}=\omega_{a}-\omega_{d}$ is the frequency detuning characterizing the atomic effective frequency in the rotating frame, and $\Delta_{0}=\omega_{c}-\omega_{d}$ denotes the detuning between the cavity mode and the driving field. Here, we have assumed that $\delta =\omega_{m}$ and used the mean-field approximations $\langle c^{†}c\rangle =\langle c^{†}\rangle \langle c\rangle$, $\langle b^{†}c\rangle =\langle b^{†}\rangle \langle c\rangle$ and 〈bc〉=〈b〉〈c〉. The steady-state average values of the system can be obtained

(6) $$\begin{array}{ccc} a_{s} & = & \frac{ig_{1}\sqrt{N}}{\gamma_{a}+i\Delta_{a}},\\ b_{s} & = & \frac{ig_{2}{\left|c_{s}\right|}^{2}}{\gamma_{m}+i\omega_{b}},\\ c_{s} & = & \frac{\varepsilon_{d}}{2\kappa +i\left(\Delta +\Delta_{F}\right)+\frac{g_{1}^{2}N}{\gamma_{a}+i\Delta_{a}}}, \end{array}$$

where the effective frequency detuning $\Delta =\omega_{c}-\omega_{d}-g_{2}\left(b_{s}^{*}+b_{s}\right)=\Delta_{0}-g_{2}\left(b_{s}^{*}+b_{s}\right)$.

In this scheme, we focus on the mean-field response of the system to the weak probe field. Under the conditions $|\varepsilon_{p}\left|\ll \right|\varepsilon_{d}|$, $|\varepsilon_{m}\left|\ll \right|\varepsilon_{d}|$, we decompose the Heisenberg operators into their steady-state mean values plus small fluctuations, i.e., $a=a_{s}+\delta a$, $b=b_{s}+\delta b$, and $c=c_{s}+\delta c$. Substituting these expansions into Equation (5), and retaining only terms to first order in the small fluctuations while neglecting nonlinear contributions, we derive the equations of motion for the expectation values of these fluctuations as

(7) $$\begin{array}{ccc} \langle \delta \dot{a}\rangle & = & -\left(\gamma_{a}+i\Delta_{a}\right)\langle \delta a\rangle +ig_{1}\sqrt{N}\langle \delta c\rangle ,\\ \langle \delta \dot{b}\rangle & = & -\left(\gamma_{m}+i\omega_{m}\right)\langle \delta b\rangle +ig_{2}\left(c_{s}^{*}\langle \delta c\rangle +c_{s}\langle \delta c^{†}\rangle \right)+\varepsilon_{m}e^{-i\delta t},\\ \langle \delta \dot{c}\rangle & = & -\left[2\kappa +i\left(\Delta_{0}+\Delta_{F}\right)\right]\langle \delta c\rangle +ig_{2}c_{s}\left(\langle \delta b\rangle +\langle \delta b^{†}\rangle \right)+\varepsilon_{d}\\ & + & \varepsilon_{p}e^{-i\delta t}+ig_{1}\sqrt{N}\langle \delta a\rangle . \end{array}$$

According to Equation (7), we use the following ansatz:

(8) $$\begin{array}{c} \langle \delta s\rangle =\delta s_{+}\varepsilon_{p}e^{-i\delta t}+\delta s_{-}\varepsilon_{p}^{*}e^{i\delta t}, \end{array}$$

where s=a,b,c. By substituting Equation (8) into Equation (7), and comparing the coefficients of $\varepsilon_{p}e^{-i\delta t}$ and $\varepsilon_{p}^{*}e^{i\delta t}$ on both sides of the equation, the solution for the fluctuations of interest can be calculated as

(9) $$\begin{array}{ccc} \langle \delta c_{+}\rangle & = & \frac{A_{-}B_{-}B_{+}-g_{2}^{2}{\left|c_{s}\right|}^{2}\left(B_{-}-B_{+}\right)}{A_{-}A_{+}B_{-}B_{+}+g_{2}^{2}{\left|c_{s}\right|}^{2}\left(A_{-}-A_{+}\right)\left(B_{-}-B_{+}\right)}\\ & + & \frac{ig_{2}c_{s}A_{-}B_{-}Re^{-i\Phi }}{A_{-}A_{+}B_{-}B_{+}+g_{2}^{2}{\left|c_{s}\right|}^{2}\left(A_{-}-A_{+}\right)\left(B_{-}-B_{+}\right)} \end{array}$$

with

(10) $$\begin{array}{ccc} A_{\pm } & = & 2\kappa -i\delta \pm i\left(\Delta +\Delta_{F}\right)+\frac{g_{1}^{2}N}{\gamma_{a}-i\delta \pm i\Delta_{a}},\\ B_{\pm } & = & \gamma_{m}-i\delta \pm i\omega_{b},\\ R & = & |\varepsilon_{m}/\varepsilon_{p}|,\;\;\;\Phi =\phi_{m}-\phi_{p}, \end{array}$$

where $A_{\pm }$ and $B_{\pm }$ are auxiliary functions that appear in the solution of the fluctuation equations, *R* is the amplitude ratio of the weak mechanical pump field to the probe field, and $\Phi =\phi_{m}-\phi_{p}$ is the phase difference between them. In Equation (9), the first term accounts for the effect of the atomic ensemble on the optomechanical system, while the second term represents the contribution from the weak mechanical pump field to the atom-assisted optomechanical system.

Employing the input-output relation [47]

(11) $$\begin{array}{c} \varepsilon_{in}\left(t\right)+\varepsilon_{out}\left(t\right)=2\kappa \langle \delta c\rangle , \end{array}$$

the output field $\varepsilon_{T}$ can be obtained as

(12) $$\begin{array}{ccc} \varepsilon_{T} & = & 2\kappa \delta c_{+}\\ & = & \mu +i\upsilon , \end{array}$$

where the real part μ=Re(εT) and imaginary part υ=Im(εT) correspond to the absorption and dispersion of the probe field in this atom-assisted hybrid system, respectively.

## 3. Discussion

**Figure 2:**
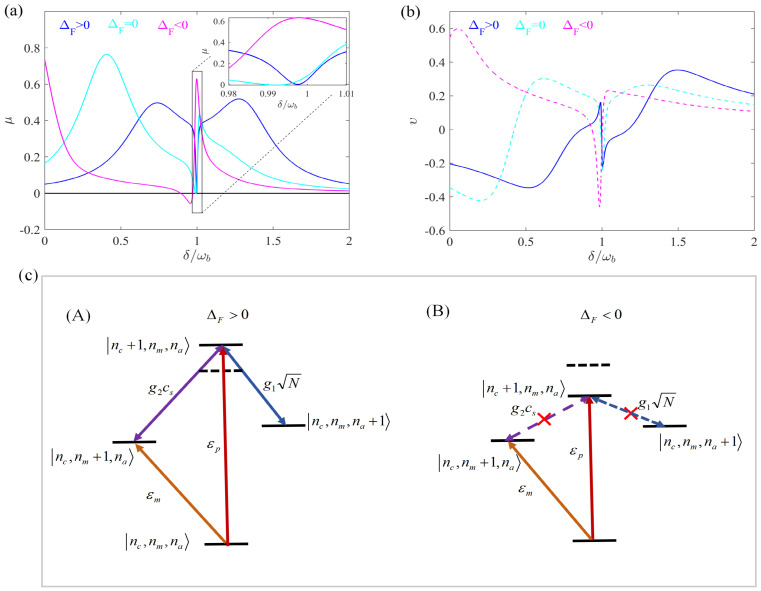
(**a**) μ (solid curve line) and (**b**) υ (dashed curve line) versus detuning $\delta /\omega_{b}$ for different $\Delta_{F}$. (**c**) Energy-level diagram for EOMIT under opposite rotation directions. The horizontal dashed line marks the $\Delta_{F}=0$ reference energy level for $|n_{c}+1,n_{m},n_{a}\rangle$. All photon-number states are shifted above this line for $\Delta_{F}>0$ (**A**) and below it for $\Delta_{F}<0$ (**B**) to represent the Sagnac–Fizeau effect. For the $\Delta_{F}<0$ case, the off-resonant OMIT and EIT pathways are marked with red crosses.

In this section, we study the absorption and dispersion spectra of the probe field in a rotating atom-assisted optomechanical system. We use the experimentally feasible parameters [44,48,49]: r=0.3 mm, n=1.44, λ=794.89 nm, $m=5\times 10^{-11}$ kg, $P_{d}=1$ mW, $\omega_{b}/2\pi =10$ MHz, $\kappa =\omega_{b}/10$, $\gamma_{a}/2\pi =2.875$ MHz, $\gamma_{m}/2\pi =100$ Hz, $g_{1}\sqrt{N}/2\pi =3$ MHz, $\Delta_{a}=\omega_{b}$, $\Delta_{0}=0.6\omega_{b}$. As shown in Figure 2, we plot the absorption and dispersion of the output field as a function of the detuning $\delta /\omega_{b}$ for different $\Delta_{F}$. Here we choose Ω=15 kHz, R=0.02 and Φ=π/2. Based on current experimental techniques, Ω has reached values on the order of GHz [50,51]. The cases $\Delta_{F}>0$, $\Delta_{F}=0$ and $\Delta_{F}<0$ correspond to Ω>0, Ω=0 and Ω<0, respectively. It is clear from Figure 2 that the absorption is zero and the steepest dispersion occurs around $\delta /\omega_{b}=0.998\approx 1$ when $\Delta_{F}>0$, where an obvious EOMIT window can be observed. However, when the WGM resonator is stationary ($\Delta_{F}=0$) or rotates in a counter-clockwise direction ($\Delta_{F}<0$), a positive absorption occurs. In particular, when $\Delta_{F}<0$, the absorption is even up to μ=0.63.

Figure 2b presents the dispersion spectrum ν as a function of the detuning $\delta /\omega_{b}$ for the same conditions as in Figure 2a. The dispersion exhibits a strong anomalous dispersion region (negative slope) around $\delta /\omega_{b}\approx 1$ for the $\Delta_{F}>0$ case (solid curve), which is characteristic of the transparency window observed in the absorption spectrum. This anomalous dispersion indicates fast light propagation and is a hallmark of quantum interference effects such as EIT and OMIT. In contrast, for the $\Delta_{F}<0$ case (dashed curve), the dispersion shows a normal dispersion region (positive slope) around the resonance point, accompanied by the absorption peak seen in Figure 2a. This transition from anomalous to normal dispersion as $\Delta_{F}$ changes from positive to negative further confirms the directional control of the optical response by the Sagnac–Fizeau shift. The steep dispersion slope for $\Delta_{F}>0$ suggests potential applications in slow and fast light manipulation, which will be explored in future work.

The variation in absorption reflects the influence of rotation-induced frequency shift in the WGM resonator on the optical response properties of the system. The physical mechanism can be explained as follows. In this scheme, we consider $\Delta_{0}=0.6\omega_{b}$. When $\Delta_{F}>0$, the effective frequency $\Delta_{0}+\Delta_{F}\approx \omega_{b}=\Delta_{a}=\omega_{m}=\delta$, the optomechanical coupling between the WGM resonator and the mechanical resonator provides the basis for interference between different quantum paths, which results in the formation of OMIT. The interaction between the optical mode and the atomic ensemble is the origin of the EIT. The weak mechanical pump field directly excites the mechanical oscillator, providing an additional interference path. Thus, when both the optomechanical coupling and the atom–cavity interaction are taken into account, together with the introduction of a weak mechanical pump field, the quantum destructive interference among these distinct pathways gives rise to the emergence of EOMIT in the hybrid atom–optomechanical system. However, the frequency shift arising from the rotation of the optical mode leads to the different optomechanical coupling and atom–cavity coupling strengths, so the different absorption spectra can be obtained.

To understand the physical mechanism behind the directional EOMIT observed in Figure 2a,b, we present in Figure 2c an energy-level diagram that illustrates the quantum interference among four distinct pathways connecting the initial state $|n_{c},n_{m},n_{a}\rangle$ to the final state $|n_{c}+1,n_{m},n_{a}\rangle$. The states are labeled as $|n_{c},n_{m},n_{a}\rangle$, where $n_{c}$, $n_{m}$, and $n_{a}$ denote the excitation numbers in the cavity, mechanical, and atomic modes, respectively. For $\Delta_{F}>0$, there are four interference paths here, leading to the generation of EOMIT. The direct path via probe field excitation follows $|n_{c},n_{m},n_{a}\rangle \to |n_{c}+1,n_{m},n_{a}\rangle$; the OMIT path via cavity-mechanical coupling $G=g_{2}c_{s}$ follows $|n_{c},n_{m},n_{a}\rangle \to |n_{c}+1,n_{m},n_{a}\rangle \to |n_{c},n_{m}+1,n_{a}\rangle \to |n_{c}+1,n_{m},n_{a}\rangle$; the EIT path via cavity-atom coupling $g_{1}\sqrt{N}$ follows $|n_{c},n_{m},n_{a}\rangle \to |n_{c}+1,n_{m},n_{a}\rangle \to |n_{c},n_{m},n_{a}+1\rangle \to |n_{c}+1,n_{m},n_{a}\rangle$; and the mechanical pump path driven by $\varepsilon_{m}e^{i\Phi }$ follows $|n_{c},n_{m},n_{a}\rangle \to |n_{c},n_{m}+1,n_{a}\rangle \to |n_{c}+1,n_{m},n_{a}\rangle \to |n_{c}+1,n_{m},n_{a}\rangle$. The horizontal dashed line marks the $\Delta_{F}=0$ reference energy level for the single-photon state $|n_{c}+1,n_{m},n_{a}\rangle$, and the energy levels of all photon-number states are shifted above this line for $\Delta_{F}>0$ and below it for $\Delta_{F}<0$ to visually represent the effect of the Sagnac–Fizeau shift. For the $\Delta_{F}<0$ case, the off-resonant OMIT and EIT pathways are marked with red crosses to indicate broken resonance conditions.

The energy-level diagram in Figure 2c provides an intuitive explanation for the directional EOMIT observed in Figure 2a,b. When $\Delta_{F}>0$ (left panel of Figure 2c), the effective cavity detuning satisfies $\Delta_{0}+\Delta_{F}\approx \omega_{b}\approx \Delta_{a}$, bringing all four pathways into resonance. This resonance condition enables complete destructive interference among the pathways, producing the transparency window seen in the solid curves of Figure 2a,b. When $\Delta_{F}<0$ (right panel of Figure 2c), the effective cavity detuning deviates from $\omega_{b}$ and $\Delta_{a}$. The OMIT and EIT pathways become off-resonant, as indicated by the red crosses, weakening their contribution to the destructive interference. This imbalance gives rise to a new interference effect, leading to the absorption peak observed in Figure 2a with μ=0.63 at $\delta /\omega_{b}\approx 1$.

**Figure 3:**
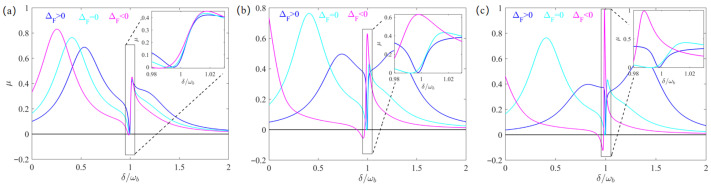
μ versus detuning $\delta /\omega_{b}$ for different $\Delta_{F}$. Here the angular velocities are set as Ω=5 kHz, Ω=15 kHz and Ω=20 kHz in (**a**), (**b**) and (**c**), respectively. All other parameters and the meanings of the three colored lines are identical to those in Figure 2.

**Figure 4:**
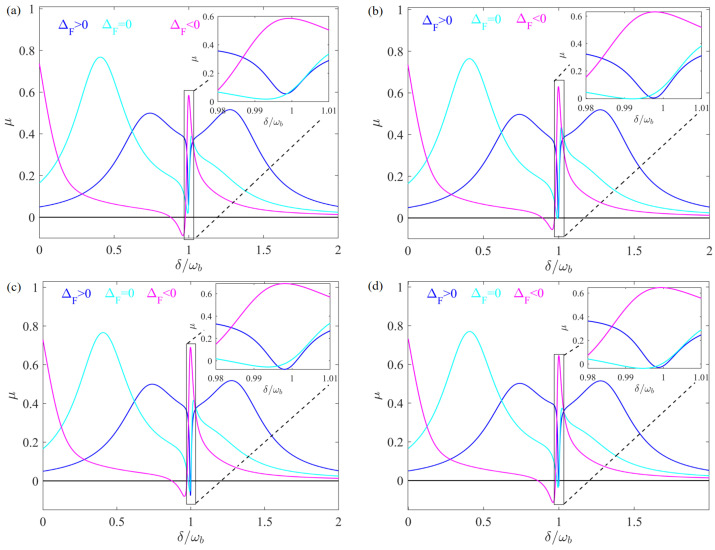
μ versus detuning $\delta /\omega_{b}$ for different Φ. The phase differences Φ are chosen as Φ=0, Φ=π/2, Φ=π, and Φ=3π/2 in (**a**), (**b**), (**c**), and (**d**), respectively. All other parameters and the meanings of the three colored lines are identical to those in Figure 2.

Before proceeding to discuss the effects of angular velocity and phase difference, it is worth addressing the origin of the gain (negative absorption) observed in Figure 2, Figure 3 and Figure 4 for the case $\Delta_{F}<0$ near $\delta /\omega_{b}=1$. This gain arises from the coherent energy transfer facilitated by the phase difference between the mechanical pump field and the probe field. Specifically, when the phase difference is appropriately tuned, the mechanical pump field provides energy to the probe field through the optomechanical interaction, resulting in amplification rather than absorption. The gain mechanism becomes direction-dependent in our system because the Sagnac-induced frequency shift modifies the effective detuning, thereby influencing the interference condition. As will be shown in Figure 4, the phase difference Φ plays a crucial role in controlling the transition between absorption and gain.

To further explore the optical response of the atom-assisted spinning WGM system, we plot the absorption spectra of the probe field versus the detuning $\delta /\omega_{b}$ for various angular velocities Ω. From Figure 3, it is shown that the absorption peak of the probe light close to the resonance point where the EOMIT occurs experiences a blue shift and the peak value increases with increasing Ω. These phenomena indicate that the optical response properties of the system can be controlled by the angular velocity of the spinning WGM oscillator. The introduction of a weak mechanical pump provides an additional path for the occurrence of quantum interference. The phase difference Φ between the weak mechanical pump and the probe field serves as an important control parameter for modulating the optical response of the atom-assisted hybrid system under mechanical driving. As shown in Figure 4, we plot the absorption spectra of the probe light versus detuning $\delta /\omega_{b}$ for different Φ. It is clear that the valley of the absorption spectra of probe light can change from positive to negative as Φ varies for both $\Delta_{F}=0$ and $\Delta_{F}>0$.

**Figure 5:**
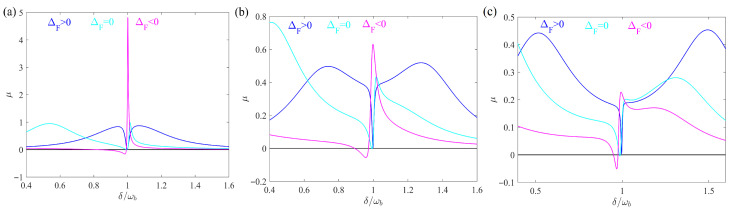
μ versus detuning $\delta /\omega_{b}$ for different $g_{1}\sqrt{N}$. Here (**a**) $g_{1}\sqrt{N}/2\pi =1$ MHz, (**b**) $g_{1}\sqrt{N}/2\pi =3$ MHz, and (**c**) $g_{1}\sqrt{N}/2\pi =5$ MHz. All other parameters and the meanings of three color lines are the same as in Figure 2.

To investigate the influence of the atomic number N on the optical response of the spinning WGM resonator system, we plot the absorption coefficient μ versus the normalized detuning $\delta /\omega_{b}$ for different values of the atom–cavity coupling strength $g_{1}\sqrt{N}$. Figure 5 shows that *N* can be used to modulate the absorption behavior of the oscillator in different rotational directions. Within a reasonable parameter range, as the number of atoms increases, the phenomenon of EMOT can always occur in the case of $\Delta_{F}>0$, while the absorption values μ of the probe field at the resonance point where EMOT occurs both decrease for $\Delta_{F}=0$ and $\Delta_{F}<0$. These results indicate that we can switch the transmission of the optical field by controlling the number of atoms and the rotation direction.

**Figure 6:**
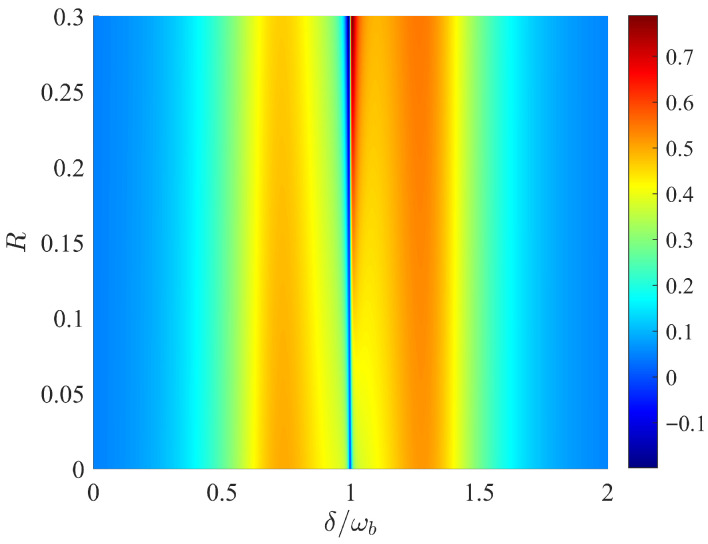
μ versus detuning $\delta /\omega_{b}$ and ratio *R*. Here we choose $\Delta_{F}>0$. All other parameters are the same as in Figure 2.

Figure 6 illustrates the influence of the introduction of the external mechanical pumping field on the optical properties of the system. As can be seen from Figure 6, when the mechanical pumping field is weak, with the increase in *R*, the depth of the OMIT window at the resonance $\delta =\omega_{b}$ gradually becomes shallower, which mainly results from the resonant absorption caused by the direct excitation of the mechanical oscillator by the mechanical pump field at position $\delta =\omega_{b}$. When the mechanical pumping field is strong enough, the resonant absorption of the mechanical oscillator also increases with the increase in the intensity of the mechanical pump field, thus causing the optomechanical effect to be completely offset. Therefore, the OMIT window disappears, and an absorption peak is gradually formed.

## 4. Conclusions

The model proposed in this scheme is experimentally feasible. First, the coupling between the mechanical oscillator and the WGM resonator can be realized experimentally by using dispersive radiation pressure. The related experimental setup, consisting of an array of nanomechanical oscillators in the form of doubly clamped SiN strings and a microcavity, has been demonstrated in Ref. [52]. Recently, experimental schemes for atom–WGM photon coupling have been proposed in Ref. [53]. The experimental approaches—either directly loading an atomic ensemble into an optical microtrap on a nanophotonic microring circuit [53], or trapping an atomic ensemble that interacts with a WGM resonator via a cycling transition above the microring resonator Ref. [54]—can ensure the experimental realization of atom–WGM photon coupling in our scheme. Inspired by Ref. [55], the rotational motion of the WGM resonator can be achieved by mounting it on a turbine and positioning the assembly adjacent to the tapered region of an optical fiber. To address the experimental feasibility of radiation pressure in our system, we provide a quantitative estimation based on the parameters in Section 2. For a rotation speed Ω=15 kHz, the Sagnac–Fizeau shift from Equation (1) is $|\Delta_{F}|\approx 4.0$ MHz. Using the standard cavity optomechanics relation $n_{c}={\left|c_{s}\right|}^{2}$ with $c_{s}$ given by Equation (6), we obtain the steady-state photon number. For $\Delta_{F}>0$, $n_{c}=1.43\times 10^{7}$, yielding a radiation pressure force $F_{\mathrm{rad}}=\hbar \omega_{c}n_{c}/r=11.9$ nN via the formula derived from the optomechanical interaction Hamiltonian [56]. For $\Delta_{F}<0$, $n_{c}=2.00\times 10^{8}$ and $F_{\mathrm{rad}}=166.3$ nN. These values are well within experimentally accessible ranges, as demonstrated by measurements of 80 fN in lightsail membranes [57]. Moreover, the effective optomechanical coupling $g_{\mathrm{eff}}=g_{2}\sqrt{n_{c}}$ reaches 0.6–2.3 MHz, three to four orders of magnitude larger than the mechanical decay rate $\gamma_{m}/2\pi =100$ Hz. These calculations confirm that the radiation pressure effects in our system are clearly observable with current technology.

In summary, we have theoretically investigated the optical response properties of an atom-assisted spinning optomechanical system. Our results demonstrate that the Sagnac effect induces non-reciprocal optical control. When the resonator rotates clockwise ($\Delta_{F}>0$), the system exhibits a transparency window with near-zero absorption (μ≈0) at resonance, while reversing the rotation direction ($\Delta_{F}<0$) leads to strong positive absorption (μ=0.63). A key finding is that the system exhibits gain (negative absorption) for $\Delta_{F}<0$ near $\delta /\omega_{b}=1$. This gain originates from the coherent energy transfer mediated by the mechanical pump field. When the phase difference is appropriately tuned, the mechanical pump field transfers energy to the probe field via constructive interference, leading to amplification. This mechanism is analogous to optomechanically induced amplification reported in previous works [18,20], and the direction-dependent features arise from the Sagnac-induced frequency shift, which modifies the effective detuning for opposite rotation directions. Furthermore, the phase difference between the mechanical pump and the probe field enables coherent switching between absorption and gain, demonstrating the tunability of this hybrid system. These non-reciprocal effects, which go significantly beyond previously reported OMIT or EIT phenomena, arise from the synergistic interplay of the spinning resonator (providing direction-dependent coupling via the Sagnac effect), the atomic ensemble (enabling EIT-based quantum interference), and the mechanically driven oscillator (introducing the phase difference as an additional control parameter).

Importantly, all control parameters, including the rotation direction, angular velocity, and phase difference, are experimentally accessible and straightforward to tune, as supported by recent experimental demonstrations [50,51,52,53,54,55]. Our results not only deepen the understanding of quantum coherence in hybrid spinning systems but also provide a theoretical foundation for developing non-reciprocal quantum devices such as optical isolators and chiral photonic components.

## Data Availability

Dataset available on request from the authors, the raw data supporting the conclusions of this article will be made available by the authors on request.

## References

[1] Harris S.E. (1997). Electromagnetically induced transparency. Phys. Today.

[2] Marangos J.P. (1998). Electromagnetically induced transparency. J. Mod. Opt..

[3] Boller K.-J., Imamoğlu A., Harris S.E. (1991). Observation of electromagnetically induced transparency. Phys. Rev. Lett..

[4] Turukhin A.V., Sudarshanam V.S., Shahriar M.S., Musser J.A., Ham B.S., Hemmer P.R. (2001). Observation of ultraslow and stored light pulses in a solid. Phys. Rev. Lett..

[5] Wu Y., Deng L. (2004). Ultraslow optical solitons in a cold four-state medium. Phys. Rev. Lett..

[6] Reiserer A., Rempe G. (2015). Cavity-based quantum networks with single atoms and optical photons. Rev. Mod. Phys..

[7] Sun C., Cui K., Chao S., Wei Y., Yuan J., Cao J., Shu H., Huang X. (2023). Sympathetic electromagnetically induced transparency ground state cooling of a ^40^Ca^+^–^27^Al^+^ pair in an 27Al+ clock. Chin. Phys. B.

[8] Zeng L.-Y., Wu J.-F., Li C. (2023). Dynamic light storage based on controllable electromagnetically induced transparency effect. Chin. Phys. B.

[9] Agarwal G.S., Huang S. (2010). Electromagnetically induced transparency in mechanical effects of light. Phys. Rev. A—At. Mol. Opt. Phys..

[10] Weis S., Rivière R., Deléglise S., Gavartin E., Arcizet O., Schliesser A., Kippenberg T.J. (2010). Optomechanically induced transparency. Science.

[11] Malykin G.B. (2021). Enhanced optomechanically induced transparency via atomic ensemble in optomechanical system. Quantum Inf. Process..

[12] Yan X.-B., Gu K.-H., Fu C.-B., Cui C.-L., Wang R., Wu J.-H. (2014). Optical switching of optomechanically induced transparency and normal mode splitting in a double-cavity system. Eur. Phys. J. D.

[13] He Y. (2015). Optomechanically induced transparency associated with steady-state entanglement. Phys. Rev. A.

[14] Zhang J.-Q., Li Y., Feng M., Xu Y. (2012). Precision measurement of electrical charge with optomechanically induced transparency. Phys. Rev. A—At. Mol. Opt. Phys..

[15] Ma P.-C., Zhang J.-Q., Xiao Y., Feng M., Zhang Z.M. (2014). Tunable double optomechanically induced transparency in an optomechanical system. Phys. Rev. A.

[16] Wang Q., Zhang J.-Q., Ma P.-C., Yao C.M., Feng M. (2015). Precision measurement of the environmental temperature by tunable double optomechanically induced transparency with a squeezed field. Phys. Rev. A.

[17] Huang S., Agarwal G.S. (2010). Normal-mode splitting and antibunching in Stokes and anti-Stokes processes in cavity optomechanics: Radiation-pressure-induced four-wave-mixing cavity optomechanics. Phys. Rev. A—At. Mol. Opt. Phys..

[18] Hao H., Kuzyk M.C., Ren J.J., Zhang F., Duan X., Zhou L., Zhang T., Gong Q., Wang H., Gu Y. (2019). Electromagnetically and optomechanically induced transparency and amplification in an atom-assisted cavity optomechanical system. Phys. Rev. A.

[19] Das S., Dey T.N. (2022). Phase-dependent controllable field generation in a ring cavity resonator. J. Opt. Soc. Am. B.

[20] Jiang C., Cui Y., Zhai Z., Yu H., Li X., Chen G. (2019). Tunable transparency and amplification in a hybrid optomechanical system with quadratic coupling. J. Phys. B At. Mol. Opt. Phys..

[21] Safavi-Naeini A.H., Alegre T.P.M., Chan J., Eichenfield M., Winger M., Lin Q., Hill J.T., Chang D.E., Painter O. (2011). Electromagnetically induced transparency and slow light with optomechanics. Nature.

[22] Zhang X.Y., Zhou Y.H., Guo Y.Q., Yi X.X. (2018). Optomechanically induced transparency in optomechanics with both linear and quadratic coupling. Phys. Rev. A.

[23] Stannigel K., Rabl P., Sørensen A.S., Zoller P., Lukin M.D. (2010). Optomechanical transducers for long-distance quantum communication. Phys. Rev. Lett..

[24] Molinares H., He B., Eremeev V. (2023). Transfer of quantum states and stationary quantum correlations in a hybrid optomechanical network. Mathematics.

[25] Zhan X.-G., Si L.-G., Zheng A.-S., Yang X. (2013). Tunable slow light in a quadratically coupled optomechanical system. J. Phys. B At. Mol. Opt. Phys..

[26] Akram M.J., Khan M.M., Saif F. (2015). Tunable fast and slow light in a hybrid optomechanical system. Phys. Rev. A.

[27] Liao Q., Xiao X., Nie W., Zhou N. (2020). Transparency and tunable slow-fast light in a hybrid cavity optomechanical system. Opt. Express.

[28] Zheng M.-H., Wang T., Wang D.-Y., Bai C.H., Zhang S., An C.S., Wang H.F. (2019). Manipulation of multi-transparency windows and fast-slow light transitions in a hybrid cavity optomechanical system. Sci. China Phys. Mech. Astron..

[29] Sarma B., Sarma A.K. (2016). Controllable optical bistability in a hybrid optomechanical system. J. Opt. Soc. Am. B.

[30] Jiang L., Yuan X., Cui Y., Chen G., Zuo F., Jiang C. (2017). Optical bistability and four-wave mixing in a hybrid optomechanical system. Phys. Lett. A.

[31] Weng M., Tian T., Wang Z. (2023). Vibration induced transparency: Simulating an optomechanical system via the cavity QED setup with a movable atom. Fundam. Res..

[32] Ghafoor F., Akram M.J., Ayaz M., Kokab H., Saif F. (2022). Atom-assisted coherent control of multiple-color mechanically induced switching. J. Phys. B At. Mol. Opt. Phys..

[33] Han C.-M., Wang X., Chen H., Li H.R. (2020). Tunable slow and fast light in an atom-assisted optomechanical system with a mechanical pump. Opt. Commun..

[34] Chen B., Shang L., Wang X.-F., Chen J.B., Xue H.B., Liu X., Zhang J. (2019). Atom-assisted second-order sideband generation in an optomechanical system with atom-cavity-resonator coupling. Phys. Rev. A.

[35] Ren Y.-L. (2022). Nonreciprocal optical–microwave entanglement in a spinning magnetic resonator. Opt. Lett..

[36] Zhang D.-W., Zheng L.-L., You C., Hu C.S., Wu Y., Lü X. (2021). Nonreciprocal chaos in a spinning optomechanical resonator. Phys. Rev. A.

[37] Gou C., Hu X. (2023). Simultaneous nonreciprocal photon blockade in two coupled spinning resonators via Sagnac-Fizeau shift and parametric amplification. Phys. Rev. A.

[38] Deng X., Zhang K.-K., Shui T., Yang W.X. (2024). Nonreciprocal unconventional magnon blockade via the Sagnac-Fizeau shift in an optomagnonic system. Phys. Rev. A.

[39] Lü H., Jiang Y.J., Wang Y.-Z., Jing H. (2017). Optomechanically induced transparency in a spinning resonator. Photonics Res..

[40] Li B.J., Huang R., Xu X.W., Miranowicz A., Jing H. (2019). Nonreciprocal unconventional photon blockade in a spinning optomechanical system. Photonics Res..

[41] Huang R., Miranowicz A., Liao J.-Q., Nori F., Jing H. (2018). Nonreciprocal photon blockade. Phys. Rev. Lett..

[42] Jiao Y.-F., Liu J.-X., Li Y., Yang R., Kuang L.M., Jing H. (2022). Nonreciprocal enhancement of remote entanglement between nonidentical mechanical oscillators. Phys. Rev. Appl..

[43] Malykin G.B. (2000). The Sagnac effect: Correct and incorrect explanations. Physics-Uspekhi.

[44] Genes C., Vitali D., Tombesi P. (2008). Emergence of atom-light-mirror entanglement inside an optical cavity. Phys. Rev. A—At. Mol. Opt. Phys..

[45] Chen X., Liu Y.-C., Peng P., Zhi Y., Xiao Y.F. (2015). Cooling of macroscopic mechanical resonators in hybrid atom-optomechanical systems. Phys. Rev. A.

[46] Bai C.-H., Wang D.-Y., Zhang S., Liu S., Wang H.F. (2019). Modulation-Based Atom-Mirror Entanglement and Mechanical Squeezing in an Unresolved-Sideband Optomechanical System. Ann. Phys..

[47] Walls D.F., Milburn G.J. (1944). Quantum Optics.

[48] Fu C.-B., Yan X.-B., Gu K.-H., Cui C.L., Wu J.H., Fu T.D. (2013). Steady-state solutions of a hybrid system involving atom-light and optomechanical interactions: Beyond the weak-cavity-field approximation. Phys. Rev. A.

[49] Guo H., Karpov M., Lucas E., Kordts A., Pfeiffer M.H., Brasch V., Lihachev G., Lobanov V.E., Gorodetsky M.L., Kippenberg T.J. (2017). Universal dynamics and deterministic switching of dissipative Kerr solitons in optical microresonators. Nat. Phys..

[50] Reimann R., Doderer M., Hebestreit E., Diehl R., Frimmer M., Windey D., Tebbenjohanns F., Novotny L. (2018). GHz Rotation of an Optically Trapped Nanoparticle in Vacuum. Phys. Rev. Lett..

[51] Ahn J., Xu Z.J., Bang J., Deng Y.H., Hoang T.M., Han Q., Ma R.M., Li T. (2018). Optically Levitated Nanodumbbell Torsion Balance and GHz Nanomechanical Rotor. Phys. Rev. Lett..

[52] Anetsberger G., Arcizet O., Unterreithmeier Q.P., Rivière R., Schliesser A., Weig E.M., Kotthaus J.P., Kippenberg T.J. (2009). Near-field cavity optomechanics with nanomechanical oscillators. Nat. Phys..

[53] Zhou X.C., Tamura H., Chang T.-H., Hung C.L. (2024). Trapped atoms and superradiance on an integrated nanophotonic microring circuit. Phys. Rev. X.

[54] Zhou X.C., Suresh D.A., Robicheaux F., Hung C.L. (2025). Selective collective emission from a dense atomic ensemble coupled to a nanophotonic resonator. arXiv.

[55] Maayani S., Dahan R., Kligerman Y., Moses E., Hassan A.U., Jing H., Nori F., Christodoulides D.N., Carmon T. (2018). Flying couplers above spinning resonators generate irreversible refraction. Nature.

[56] Aspelmeyer M., Kippenberg T.J., Marquardt F. (2014). Cavity optomechanics. Rev. Mod. Phys..

[57] Michaeli L., Gao R., Kelzenberg M.D., Hail C.U., Merkt A., Sader J.E., Atwater H.A. (2025). Direct radiation pressure measurements for lightsail membranes. Nat. Photonics.

